# Factors associated with stress among pregnant women with a second child in Hunan province under China’s two-child policy: a mixed-method study

**DOI:** 10.1186/s12888-024-05604-7

**Published:** 2024-02-22

**Authors:** Lijuan Zhang, Ruirui Huang, Jun Lei, Yongrong Liu, Dan Liu

**Affiliations:** 1 Department of Pediatrics, The Third Xiangya Hospital, Central South University, 410013 Changsha, Hunan China; 2 Nursing Department, The Third Xiangya Hospital, Central South University, 410013 Changsha, Hunan China; 3 School of Nursing, Hunan University of Medicine, Huaihua, Hunan China; 4 Department of Obstetrics and Gynecology, The Third Xiangya Hospital, Central South University, 410013 Changsha, Hunan China; 5 Department of Operating room, The Third Xiangya Hospital, Central South University, Changsha, Hunan China; 6 Department of Urology, The Third Xiangya Hospital, Central South University, Changsha, Hunan China

**Keywords:** Pregnancy stress, China’s two-child policy, Mixed methods, Factors

## Abstract

**Objective:**

The purpose of the study was to investigate the status of pregnancy stress and to explore factors associated with pregnancy stress among women by China’s two-child policy.

**Methods:**

A mixed-method study involving both quantitative and qualitative methods was conducted using questionnaires and semi-structured interviews. The questionnaires encompassed socio-demographic and obstetric characteristics, as well as the Pregnancy Stress Rating Scale (PSRS) and the Social Support Rating Scale (SSRS). Initially, the participants were required to complete the questionnaires, enabling us to assess their respective pregnancy stress statuses. Subsequently, we selectively interviewed pregnant women with a second child and exhibited at least mild pregnancy stress. The qualitative study sought to uncover the factors contributing to their stress during pregnancy.

**Results:**

A total of 463 subjects were enrolled; of the subjects, 22 (4.8%) had no stress, 407 (87.9%) had mild stress, 34 (7.3%) had moderate stress. Generalized linear regression analysis revealed significant factors (*P*<0.05) related to pregnancy stress, including family financial burden, subjective support, fertility desire, gender of the first child, and gender preference. Additionally, 16 subjects were interviewed, and through analysis, three major themes emerged, each comprising 12 sub-themes associated with pregnancy stress. These themes were identified as fertility factors (worry about maternal and child health, birth experience, and parenting stress), family factors ( financial burden, second child care problems, first child’s acceptance of the second child, family concerns, fertility desire, and gender preference) and social factors (involving life events, career development and workload).

**Conclusion:**

The diver factors contribute to pregnancy stress among pregnant women under China’s two-child policy. Our study could be used to develop appropriate interventions to reduce pregnancy stress and to enhance the mental health of women pregnant with a second child.

## Introduction

Pregnancy is a potentially stressful event. Pregnancy stress has been found to be prevalent during pregnancy and may adversely affect maternal and fetal health. It has been reported that up to 36.1% of women experience a certain level of stress during pregnancy [1]. Research has shown that 6.0-16.7% of pregnant women report high levels of perceived stress, and 13.6-91.86% report mild to moderate levels of perceived stress [1–6]. Maternal pregnancy stress has been linked to adverse birth outcomes [7], including preterm birth and low-birth-weight infants [8, 9]. Several studies have demonstrated that stress during pregnancy is associated with an increased risk of antenatal/postpartum depression or anxiety symptoms [10, 11] and results in increased cortisol and reduced food intake in mothers [12]. In addition, it is reasonable to consider pregnancy stress as a risk factor for infants’ development [13]. Kinga et al.’s cohort study of 372 mother-child pairs showed that pregnancy stress is significantly associated with decreased child cognition [14], and pregnancy stress relates to the cortical structure and mood in young adulthood. Some evidence suggests that higher stress during pregnancy predicts more mood dysregulation in young adulthood, lower cortical GM volume and regional GM volume in the mid-dorsolateral frontal cortex, anterior cingulate cortex and precuneus, the development of anxiety, and lower productive and receptive language abilities [15–19].

On January 1, 2016, China’s one-child policy was replaced by China’s two-child policy (a policy allowing a couple to have two children). According to official estimates, there are approximately 90 million couples in line with China’s two-child policy; 60% of women are older than 35 years of age, and 50% are at least 40 years old [20]. According to a survey on the maternal health care needs of women of childbearing age in Beijing, more than 25% of women have given birth to a second child at age 35 or older [21]. High-age pregnant women might face particular difficulties. Evidence has shown that pregnant women aged 30 years or older exhibit a significantly increased risk of uterine scarring, uterine rupture, preeclampsia, placenta damage and postpartum hemorrhage [22–27]. In addition, there are other challenges, such as high reproductive costs, which can cause a heavy financial burden, including conflict between taking care of the second child and career development. These difficulties and challenges may increase pregnancy stress, which has a negative effect on the physical and mental health outcomes of women and fetuses. Therefore, the stress and its associated factors among pregnant women with a second child deserves our focus to expore under China’s two-child policy.

It is therefore important to examine the influencing factors of pregnancy stress and explore the relationship between the influencing factors and pregnancy stress. Previous studies have examined the influencing factors of pregnancy stress and among Chinese women or women in other countries. The commonly reported factors associated with pregnancy stress in the literature include three aspects [28–30]: (1) socio-demographic aspects such as age, educational level, marital status, employment, income, relationship with husband. (2) obstetric factors. (weeks of gestation, planned pregnancy, history of abnormal gestation, etc.) (3) family and social factors, such as the level of social support and family relationships, have also been identified. The results of the research indicate a negative correlation between social support and pregnancy stress. The less social support pregnant women receive during pregnancy, the higher the level of pregnancy stress they experience. Low social support plays a crucial predictive role in severe pregnancy stress. It can account for 50% of the total variability in pregnancy stress among pregnant women. However, subject to regional cultural influences, variations in sample size, the inclusion of pregnant women from different gestational periods, diverse childbirth experiences, and various provinces or regions and other potential factors, ongoing debates persist regarding specific socio-demographic and obstetric factors associated with pregnancy stress.

To our knowledge, there have been limited studies conducted on pregnancy stress among women expecting their second child in Hunan province, China. Furthermore, almost all of these studies have primarily utilized quantitative research methods [1–6, 28–30], which may not fully capture the depth and characteristics of pregnancy stress experienced by women with a second child. In this study, we employed a mixed method approach to gain a comprehensive understanding of the stress experienced by pregnant women with a second child in Hunan province, China.

Therefore, this paper aims to investigate the level of stress among pregnant women with a second child under China’s two-child policy and to explore factors associated with pregnancy stress among them by using both qualitative and quantitative methods. Results of this study can guide the development of targeted interventions and improve mental health of pregnant women with a second child.

## Methods

### Participants and settings

The participants of this study were a sample of “two-child” pregnant women receiving routine prenatal care at the obstetrical outpatient department of the Third Xiangya Hospital in Changsha Province between November 2016 and October 2017. These participants were selected by convenience sampling. Eligible criteria were (1) women pregnant with a second child and (2) single fetus and ultrasound showing no abnormalities or defects. Pregnant women with a history of psychiatric disorders or those taking psychotropic drug therapy were excluded.

The women completed self-report questionnaires in approximately 7–10 min while waiting for a routine antenatal check-up. A total of 492 questionnaires were distributed to pregnant women who met the study criteria, with 463 (94.12%) women responding to the questionnaire. They completed a structured questionnaire including socio-demographics, the Pregnancy Stress Rating Scale, and the Social Support Rating Scale. According to the findings of the quantitative study, pregnant women experiencing mild pregnancy stress or higher were recruited as interview subjects through a convenient sampling method on a voluntary basis. The semi-structured interviews were conducted by a qualified interviewer in an obstetric health education classroom in the hospital. Guided by the outline of the semi-structured interviews and based on the “sufficiency” and “saturation” principle, we stopped recruiting interviewees once the 16 interviewees were unable to provide additional new information and themes. Participants were assured that despite entering into the study, they could withdraw any time they wished, and their information would be kept confidential. Informed consent was obtained from all the pregnant women. Ethical approval for the study was obtained from Institutional Review Board of behavioral and nursing research in School of Nursing of Central South of University, China, and the protocol number was 2,017,045.

### Instruments

A quantitative assessment was performed using a set of questionnaires among pregnant women to collect quantitative data, including the Socio-demographic and Obstetric Characteristics Questionnaire, the Pregnancy Stress Rating Scale (PSRS) and the Social Support Rating Scale (SSRS). A semi-structured interview outline was used to guide the in-depth interviews to collect qualitative data.

#### Socio-demographic and Obstetric Characteristics Questionnaire

This questionnaire included two aspects, namely, socio-demographic and obstetric data. Socio-demographic data included nationality, residence, age, educational level, occupation, monthly income (in Chinese currency, Yuan), relationship with husband, relationship with parents-in-law, family financial burden, fertility desire, gender of first child, and gender preference. Obstetrical data included weeks of gestation, planned pregnancy, history of abnormal gestation, pregnancy complications, mode of first delivery, and mode of second delivery.

#### Pregnancy Stress Rating Scale (PSRS)

The Chinese version the PSRS was developed by Chen and colleagues [31] in Taiwan, China, in 1983. The scale measures the source and level of stress unique to the pregnancy experience, including potentially stressful pregnancy-related events and anticipated events during the labor, delivery, and immediate postpartum periods. It has been widely used among Chinese women to assess stress during pregnancy. The scale contains 30 items and is categorized into four factors: factor 1 “parenthood recognition” (15 items), factor 2 “assurance of the health and safety of mother and fetus” (8 items), factor 3 “changes of body shape and physical activities of mother” (4 items), and factor 4 “other” (3 items). The three items included in “other” were “concern about the ability to rear child properly”, “concern about spouses’ mutual affection after having a baby”, and “concern about inability to provide the child with good support”. The PSRS is a Likert-style scale (4-point): 0 means “no stress”, 1 means “mild stress”, 2 means “moderate stress”, and 3 means “high stress”. All items’ scores were added to create a total stress score, with a higher score indicating greater pregnancy stress. The original total score ranges from 0 to 90. Dividing the original total score by 30 produces the standardized score (0 indicates “no stress”, 0.001～1.000 indicates “mild stress”, 1.001～2.000 indicates “moderate stress”, and 2.001～3.000 indicates “high stress”). The internal consistency by Cronbach’s alpha of the PSRS was 0.92 [32].

#### Social Support Rating Scale (SSRS)

Social support was measured by the Chinese version of SSRS. It was originally developed in China by Xiao Shuiyuan in 1986 [33]. The SSRS contains 3 dimensions and 10 items. The dimensions are objective support (3 items), subjective support (4 items), and availability of social support (3 items). Adding the scores of all items produces a total score. A higher score indicates greater social support. The SSRS has been widely used in assessing social support in Chinese populations. This scale’s Cronbach’s alpha coefficient was 0.89 among Chinese college students [34], the two-month test-retest reliability coefficient was 0.92 [35], and the reliability coefficient of each item was 0.89 ~ 0.94 [33].

#### Semi-structured interview outline

The interviews were conducted by a qualified interviewer. Face-to-face interviews were conducted and were guided by a semi-structured interview outline. The outline included 5 open-ended questions in the following order: (1) Do you worry about this pregnancy? (2) What are your concerns? (3) Why do you have this worry? (4) Who do you tell this worry? (5) Does your concern affect you? Each interview lasted between 30 and 45 min.

### Data Analysis

The quantitative data were entered into EpiData 3.1 and analyzed using IBM SPSRS v22.0 statistical software for Windows. Qualitative variables were presented as frequency, percentage, median, and quartile. Non-normally distributed data sets were compared using the Mann-Whitney U test, while multiple data sets were compared using the Kruskal-Wallis H test. The relationship between social support and pregnancy stress was examined through Spearman correlation tests. A generalized linear regression model was utilized for multifactorial analysis. The analyses were two-tailed, and statistical significance was considered at *P* < 0.05. To ensure patient privacy and facilitate data organization and analysis after the interviews, each interviewee was assigned a number (e.g., N5 for Number 5). Within 24 h of the interview, the audio recordings were transcribed verbatim into Word documents, and thematic analysis was employed for analyzing the qualitative data, including familiarization, coding, interpretation of findings, and summarization of major themes [36].

## Results

### Quantitative results

A total of 463 pregnant women were recruited for this study, in accordance with the universal two-child policy. The prevalence of pregnancy stress was observed in this study, with 407 (87.9%) pregnant women experiencing mild stress and 34 (7.3%) experiencing moderate stress. The median pregnancy stress total score for the study population was 0.367 (Quartile = 0.4). The majority of the pregnant women (93.3%) were of Han nationality. Furthermore, 78.4% of the participants resided in inner city areas, while 14.5% were from towns and 7.1% came from rural areas. It is worth noting that 99.8% of the pregnant women were married, and the majority (98.5%) had conceived naturally, with only 1.5% undergoing assisted reproductive techniques. The distribution of pregnancy stress is presented in Table 1 below.

**Table 1:** The median scores with total and four dimensions of pregnancy stress

pregnancy stress	Median	Quartile
Total score	0.367	0.400
Factor 1	0.200	0.333
Factor 2	0.625	0.750
Factor 3	0.250	1.000
Other	0.333	0.667

Note: factor 1=“parenthood recognition” (15 items), factor 2=“assurance of the health and safety of mother and fetus” (8 items), factor 3=“changes of body shape and physical activities of mother” (4 items), and factor 4=“other”

Based on the findings from Table 2, significant differences (*P* < 0.05) were observed in the PSRS scores among pregnant women based on their age, gestational weeks, educational levels, employment status, marital relationships, relationships with their mother-in-law, planned pregnancies, gender of their first child, fertility desire, and family financial burden. Additionally, Table 3 reveals significant correlations (*P* < 0.01) between the total PSRS score and the total SSRS score (*r* = -0.227), as well as the subjective support score (*r* = -0.227) and social support score (*r* = -0.092).

**Table 2:** Univariate analysis of socio-demographic and obstetrical characteristics for pregnancy stress

Group	N(%)	Median	Z/χ^2^	*P*
**Age**				
< 35	314(67.8)	0.367	-0.202^*^	0.840
≥ 35	149(32.2)	0.367		
**Weeks of gestation**			2.544	0.280
≤ 12	93(20.1)	0.333		
12–28	204(44.1)	0.317		
> 28	166(35.9)	0.367		
**Educational level**			-2.538^*^	0.011
college or above	218(47.1)	0.383		
junior college or lower	245(52.9)	0.300		
**Employment**			11.327	0.125
civil servant	23(5.0)	0.300		
Teacher	58(12.5)	0.383		
medical worker	21(4.5)	0.700		
Workers	16(3.5)	0.350		
Farmers	4(0.9)	0.300		
individual worker	89(19.2)	0.400		
unemployed	51(11.0)	0.300		
Other	201(43.4)	0.333		
**Monthly income (RMB)**			0.341	0.952
< 2000	18(3.9)	0.333		
2000 ~ 4000	94(20.3)	0.367		
4000 ~ 6000	137(29.6)	0.367		
≥ 6000	214(46.2)	0.333		
**Relationship with husband**			3.016	0.082
Good	455(98.3)	0.333		
fair	8(1.7)	0.667		
poor	0(0)	0		
**Relationship with mother-in-law**			11.146	0.004
good	391(84.4)	0.333		
fair	70(15.1)	0.433		
poor	2(0.4)	0.917		
**Planned pregnancy**			-2.370^*^	0.018
yes	357(77.1)	0.333		
no	106(22.9)	0.433		
**History of abnormal gestation**			-2.360^*^	0.018
yes	143(30.9)	0.400		
no	320(69.1)	0.316		
**Pregnancy complications**			0.460^*^	0.645
yes	93(20.1)	0.367		
no	370(79.9)	0.350		
**Gender of first child**			-2.001^*^	0.045
male	259(55.9)	0.333		
female	204(44.1)	0.367		
**Mode of first delivery**			-0.971^*^	0.331
spontaneous vaginal	255(55.1)	0.300		
caesarean	208(44.9)	0.367		
**Fertility desire**			-3.976^*^	0.000
voluntary	291(62.9)	0.300		
involuntary	172(37.1)	0.433		
**Gender preference**			6.126	0.047
male	57(12.3)	0.433		
female	164(35.4)	0.383		
doesn’t matter	242(52.3)	0.300		
**Mode of second delivery**			1.284	0.526
spontaneous vaginal	288(62.2)	0.350		
caesarean	122(26.3)	0.367		
uncertain	53(11.4)	0.367		
**Family financial burden**			31.907	0.000
unbearable	11(2.4)	0.350		
basic allowed	326(70.4)	0.367		
completely allowed	126(27.2)	0.367		

Note: “^*^”: Z

**Table 3:** Spearman correlation between PSRS and SSRS

	The total score of pregnancy stress
The total score of social support	-0.227^**^
Scores of subjective support	-0.227^**^
Scores of objective support	-0.088
Scores of availability of support	-0.092^**^

Note: ^**^p < 0.01

As shown in Table 4, the pregnancy stress score follows a skewed distribution, and after logarithmic conversion, it still exhibits skewness. Therefore, Generalized Linear Model (GLM) analysis is employed for the regression analysis. Following Univariate analysis and Spearman correlation analysis, several factors were found to have statistical significance (*P* < 0.05): educational level, relationship with mother-in-law, planned pregnancy, history of abnormal gestation, gender of first child, fertility desire, gender preference, family financial burden, subjective support, and availability of support. These factors were included in the generalized linear regression model. The GLM revealed that the likelihood ratio test was χ^2^ = 111.279, v = 16, *P* < 0.001, indicating that the GLM statistical significance.

**Table 4:** Generalized linear regression analysis for pregnancy stress in pregnant women

Covariate	B	SE	Wald χ^2^	df	*P*
Family financial burden					
unbearable	0.790	0.212	13.828	1	0.000
basic allowed	0.341	0.073	21.546	1	0.000
completely allowed	reference				
Subjective support	-0.023	0.009	7.115	1	0.008
Fertility desire					
voluntary	-0.271	0.066	17.000	1	0.000
involuntary	reference				
Gender of first child					
male	-0.288	0.082	12.439	1	0.000
female	reference				
Gender preference					
male	0.140	0.101	1.923	1	0.166
female	0.260	0.082	10.017	1	0.002
doesn’t matter	reference				

Note: B = standardized regression coefficient, SE = standard error, df = Degrees of Freedom

The variables included in the GLM were family financial burden, subjective support, fertility desire, gender of first child, and gender preference. The B value is utilized to assess how strongly various independent variables influence the dependent variable. Typically, when there is statistical significance, a larger absolute value for B indicates a greater impact of the respective independent variable on the dependent variable. Specifically, pregnancy stress of unbearable and basic allowed family financial burdens were 0.790 points 0.341 points higher than those of the reference group, respectively. Subjective support, voluntary, gender of first child is male are protective factors for pregnancy stress. Pregnant women who expressed gender preference of a girl exhibited a significantly higher score of 0.260 in terms of pregnancy stress compared to those without any specific gender preferences.

### Qualitative results

A total of 16 women pregnant with a second child participated in the in-depth interviews. Of the participants, 43.75% (*N* = 7) had a college education or above. On average, the pregnant women were 32.63 years old. Three themes related to pregnancy stress were identified: fertility factors, family factors and social factors (Table 5).

**Table 5:** Themes and sub-themes of influencing factors of pregnancy stress in pregnant women with two children

Themes	Sub-themes
Fertility factors	Worry about maternal and child health
	Birth experience
	Parenting stress
Family factors	Financial burden
	Second child care problems
	First child’s acceptance of the second child
	Family concerns
	Fertility desire
	Gender preference
Social factors	Life events
	Career development Workload

### Fertility factors

#### Worry about maternal and child health

Four women who were pregnant with a second child expressed concerns about their declining physical function and increased pregnancy complications, primarily due to their advanced maternal age (three of them were over 35 years old). Consequently, both the mother and fetus experienced poor physical health during the pregnancy, adding to the mother’s burden of pregnancy stress.

*“My primary source of stress stems from my advancing age. It is frequently reported in the media that older mothers have a higher likelihood of giving birth to babies with congenital abnormalities. This concern deeply troubles me as it represents my greatest worry.” (Interviewee N3)*.

#### Birth experience

Four expectant mothers, in anticipation of their second child, expressed lingering negative memories of their initial childbirth experience. They harbor apprehensions about the likelihood of encountering a similar adverse encounter during their second delivery.

*“My initial delivery was prolonged, with the second stage of labor lasting over 3 hours. From the onset of labor to the birth of my baby, the entire process lasted for more than eight arduous hours, causing intense discomfort. Administering oxytocic proved highly effective, swiftly alleviating the pain. It took a grueling eight hours to bring forth my child, hence, I now harbor stress towards this upcoming delivery as well (Interviewee N13).”*

#### Parenting stress

Six expectant mothers voiced their exhaustion in caring for their initial offspring, expressing their reluctance to undertake the toilsome responsibilities of childcare anew.

*I faced challenges in raising my first child, and I anticipate experiencing them again with a second child. Additionally, I must simultaneously care for my first child, which adds to my stress and occasionally even induces fear. While managing one child was already demanding, the responsibility of looking after two after giving birth will undoubtedly exhaust me before my second child reaches the age of 2 (Interviewee N5).*

### Family factors

#### Financial burden

Twelve expectant mothers, who were already mothers to one child, attributed their pregnancy stress to economic issues, which were mentioned a staggering 45 times. The arrival of their second child meant a transition in their family structure, changing from a “421” to a “422” arrangement. This placed the couple in the predicament of the “sandwich mezzanine”, wherein they found themselves tasked not only with raising their two children at considerable expense, but also providing financial support for their four aging parents with substantial pension costs.

*“Stress is a result of both our parental and filial responsibilities. When unfortunate events occur simultaneously in both areas, it becomes exceedingly challenging to handle. Moreover, as a parent, I often have to contend with the occasional illness of my two children, all while balancing my time and energy for my job. Consequently, stress permeates every aspect of my life, particularly when work-related matters intensify. The confluence of pressures from parents, children, and work exacerbates the overall stress levels.” (Interviewee N5)*.

#### Second child care problems

Five expectant mothers, anticipating the arrival of their second child, have expressed concerns regarding the ability of their aging in-laws to adequately attend to two children. These women harbor apprehensions about the capacity of their parents-in-law, who have advanced in years, to effectively care for an additional offspring.

*“My parents-in-law, who are in their 70s, find it increasingly difficult to care for my two children, so I am solely responsible for their upbringing. While my first child was previously looked after by my parents, I personally lack any prior experience in parenting. This presents a significant challenge for me, leaving me deeply concerned.” (Interviewee N15)*.

#### First child’s acceptance of the second child

Pregnant women often experience challenges as their first child may display resistance towards the impending arrival. This situation can evoke instability and unease for the expectant mother. However, it is crucial to recognize that such emotional instability can negatively impact the physical and mental well-being of the firstborn. Hence, it is essential for pregnant women and their families to prioritize the needs of their first child, thereby fostering a harmonious environment conducive to their development.

*“I informed the first child of my pregnancy, sharing the news that a younger brother or sister would soon be joining our family. Upon hearing this, he immediately became infuriated and lost control of his emotions, uttering threatening remarks like, ‘I wish to harm the newcomer,’ and engaging in unacceptable behavior.” (Interviewee N4).*

#### Family concerns

The family is the most basic and important living unit. The psychological status and stress of family members are deeply affected by the degree of concern among family members. According to the interviews, compared with the first child, the concern from their husbands or parents-in-law was reduced, which increased the psychological stress of these pregnant women.

*“He (husband) has not accompanied me every day like when I was pregnant the first time. He lets me go to the hospital myself and sometimes gives me some money. He doesn’t feel surprised and excited like the first child, and he doesn’t treat me like a fragile treasure” (Interviewee N14).*

#### Fertility desire

Some pregnant women have expressed their unwillingness to have a second child, yet they are pressured by their husbands and other family members to maintain family harmony. This involuntary childbirth not only leaves them feeling disrespected but also subjects them to poor psychological well-being and significant stress during pregnancy. These women are excessively concerned about the potential negative impact of this stress on the growth and development of their unborn child.

*“I now have a daughter, but I am not keen on having a second child. However, my husband and his parents persistently advised me to have another one. Initially, I disagreed with their suggestion. This pregnancy was unexpected. Although my husband desired it, I did not share the same sentiment. Upon discovering my pregnancy, I found myself constantly in tears, avoiding conversations. I harbor anger towards my husband and his family, and I am struggling to come to terms with this situation” (Interviewee N3).*

#### Gender preference

“I desire a daughter because raising girls is relatively effortless. However, my parents-in-law have a strong preference for a son, particularly my father-in-law. This sentiment is shared by my husband as well. He has expressed to me that a son would be our own, while a daughter would eventually belong to someone else as a bride. If I were to give birth to a girl, contrary to their expectations, their disappointment would be profound. Therefore, I am currently under immense stress.” (Interviewee N6).

*“I desire to have a daughter as I believe girls are easier to raise. Conversely, my in-laws strongly desire a son; particularly my father-in-law, as does my husband. My husband has conveyed to me that a son would be our direct descendant, whereas a daughter would belong to someone else’s family as a bride. If I were to give birth to a girl, contrary to their expectations, it would greatly disappoint them. Consequently, you can understand the immense stress I am under.” (Interviewee N6)*.

### Social factors

#### Life events

Life events encompass various changes that individuals encounter in their daily lives, such as a decline in affection within couples, enrolling in school, and family members falling ill. During interviews, some expectant mothers mentioned the stress triggered by life events during pregnancy. Among them, one pregnant woman experienced a trifecta of life events simultaneously: her first child’s enrollment in school, her husband’s company going bankrupt, and her mother falling seriously ill. The greater the number of life events a pregnant woman undergoes, the more profound the impact on her physical and mental well-being, and the more burdensome her pregnancy stress becomes.

*“My mother had just found out that she had cancer, and my husband’s own company was closed down and went into liquidation. My first child is three years old, and I have to find a school at the beginning of October for him. I am very tired. The stress from many aspects is very great. All of them came together, all unexpected; that wasn’t what we expected (crying)” (Interviewee N5).*

#### Career development

According to the results of the quantitative research, the average age of the pregnant women was approximately 32.57 years. Most of them are the backbone of the work unit and are in the rising period of their careers, with opportunities for various kinds of learning and promotion. However, from pregnancy to childbirth, their work units may worry that the pregnant women’s overall condition does not qualify them for their current job, which may cause these pregnant women to worry about their career development.

*“If I don’t go back to work earlier, I may be replaced” (Interviewee N4).*

*“I also think that though you don’t resign after pregnancy, you can’t go up in some important positions and you can’t move forward. It’s going to affect your career development” (Interviewee N16).*

#### Workload

Compared with other occupations, medical workers have a heavier workload, which leads to greater psychological stress. Due to the special nature of this work, such as a heavy workload, high risk factors, frequent late-night shifts, and contradictions between doctors and patients, medical workers often face physical incapacity and physical and mental stress.

*“There must be stress at work. We are nurses under stress. We are also very tired when going to work” (Interviewee N8).*

*“I’ve always thought that my career has caused my physical health to deteriorate. I often need to work on the night shift; it has been about 10 years like this, from 2005 to the present. It was particularly evident that my tolerance ability was declining. I couldn’t lift my legs when I was on the night shift, and because of this situation, I have to go to see the doctor” (Interviewee N10).*

## Discussion

In this research, pregnant women who fell under China’s two-child policy generally exhibited mild to moderate levels of pregnancy stress, the findings of this study align with previous research conducted in China [37–40]. The mild pregnancy stress may be due to 77.1% of the pregnant women experiencing planned pregnancies. The proportion exceeded the number of women experiencing unplanned pregnancies, indicating that a majority of pregnant women adhering to China’s two-child policy have diligently prepared themselves psychologically, physiologically, and materially before conceiving, enabling them to navigate various challenges during pregnancy with grace and ease. In our study, 67.8% of pregnant women were below the age of 35, while 69.1% had no history of abnormal gestation. Moreover, 79.9% did not experience any complications during pregnancy. The majority of these women and their fetuses were in good health, resulting in lower pregnancy risks and reduced stress levels. Our findings also indicate that factor 2, “assurance of the health and safety of the mother and fetus” (8 items), significantly contributes to pregnancy stress, which is similar to the results of other researchers [41]. The qualitative findings revealed that under China’s two-child policy, certain expectant mothers expressed concerns regarding advanced maternal age, reduced physical function, pregnancy complications, adverse birth experiences, and the potential negative impact on the health of their first child. Therefore, it is imperative for medical professionals to be attentive to the stress experienced by older women during pregnancy, and to enhance timely interventions aimed at alleviating their stress levels.

Both quantitative and qualitative findings have identified common factors associated with the pregnancy stress of women expecting their second child. These factors include family financial burden, subjective support, fertility desire, gender of the first child, and gender preference. The study conducted by Kavanaugh and Bloom supports the notion that women’s pregnancy stress is influenced by their family’s financial burden [41–45]. Our qualitative study further explains this result. It reveals that many couples with second children in China face the challenge of being part of the “sandwich generation” - adults who simultaneously care for their own children and aging parents. The financial burden on these individuals is even greater, leading to increased pregnancy stress. It is recommended that it is important to implement welfare measures, such as a second childbearing subsidy, to provide financial support for women with two children.

Secondly, there exists a direct correlation between a higher score in subjective support and a reduced level of stress during pregnancy, aligning with Kingston’s findings [1]. Subjective support [33] is intimately intertwined with an individual’s personal feelings, encompassing their experiences and contentment with understanding, respect, and assistance in social interactions. Thoits [46] highlighted that subjective support holds more significance than objective support. Although perceived support is not an objective reality, “the perceived reality is the psychological reality”, and it is in society that psychological reality affects people as a real variable. Analyzing the interview data revealed that family members may overlook the physical and mental changes experienced by pregnant women under China’s two-child policy, potentially attributing their experiences during pregnancy and childbirth. Consequently, pregnant women may subjectively perceive insufficient care and support from their families, leading to an increase in pregnancy-related stress. Therefore, it is crucial for family members to recognize and prioritize the physical and psychological changes endured by pregnant women, ensuring they provide comprehensive care, support, and subjective assistance to alleviate their pregnancy stress.

Thirdly, the results of the study showed that pregnant women with voluntary pregnancies had lower pregnancy stress by 0.271 points (*P* < 0.05) compared with pregnant women with involuntary pregnancies. The results of the qualitative study and the quantitative studies mutually corroborated each other’s results, consistent with previous research [38]. Several women with involuntarily pregnancy expressed that adherence to traditional Chinese beliefs, such as “more sons/children equate to greater blessings/happiness,” “having both a daughter and a son,” and “continuing the family lineage,” compelled them to have a second child under the fertility policy. In order to maintain family harmony, women were coerced into choosing fertility. During the qualitative interviews, it was observed that pregnant women who did not wish to give birth often experienced profound depression, silent tears, and hesitated to communicate with other family members. Therefore, family members should proactively take measures to alleviate familial tension, provide extensive support and understanding to pregnant women, and minimize the stress associated with involuntary fertility.

Fourth, the influence of having a female first child on pregnancy stress is evident in both quantitative and qualitative studies. Although the perception of gender roles in China has evolved, there are still numerous families who hold onto traditional beliefs. Some expectant mothers reported that if their first child was a girl, their families strongly desired a boy as a second child. This could be attributed to their husbands considering the boy as their own and the girl as someone else’s, or the influence of parents-in-law who adhere to the traditional Chinese notions of “having both a daughter and a son” and “continuing the family lineage.” As a result, pregnant women anticipating their second child experience substantial stress. Conversely, if the first child was a boy, pregnant women face fewer expectations regarding the gender of their second child from family members. It is crucial to promote healthy fertility concepts and gradually diminish the traditional Chinese ideology of “continuing the family lineage.”

Fifth, pregnant women who expressed a preference for having girls experienced a 0.26-point decrease in pregnancy-related stress (*P* < 0.05) compared to pregnant women who did not have a preference for the gender of their child. The qualitative data obtained from this study further supports this finding. Pregnant women emphasized that if their first child was a boy and they were expecting a second child, it would be more costly to raise two boys, hence their strong desire for a girl as the second child. Additionally, they mentioned that girls are generally easier to care for and gentle in nature. This sentiment aligns with an old Chinese saying that compares a daughter to a warm and comforting cotton-wadded jacket for parents. Conversely, pregnant women without a preference for the gender of their child explained that they believed it was best to simply accept whatever the outcome may be. Whether they had a son or a daughter did not matter to them as much as the overall health of the child.

In addition to the aforementioned five influencing factors, the qualitative study’s thematic analysis revealed that birth experience, parenting stress, second child care problems, first child’s acceptance of the second child, family concerns, life events, career development and workload are related to pregnancy stress.

One of the themes in the qualitative analysis of fertility factors was the birth experience. This included a low sense of the surgeon’s responsibility during the first birth, as well as prolonged duration and pain during the second labor. Women who were pregnant with their second child expressed fear of a similar negative birth experience, which resulted in varying levels of pregnancy stress. Additionally, the theme of fertility factors mentioned pregnancy stress arising from pregnant women taking care of their first child, while their husbands and other family members were less involved in the child-rearing process. This added hardship to the women’s experience. Previous study [47] have found that husbands’ support has greater predictive value for pregnancy stress compared to support from relatives and friends. In traditional Chinese culture, parenting has traditionally been viewed as solely the mother’s responsibility. However, research has demonstrated [48] that husbands’ participation in parenting plays an important role in reducing maternal parenting stress.

Family factors were identified as one of the key themes in the qualitative study that contributed to the stress experienced by women during their second pregnancy. Among these factors, the issue of second child care emerged as a particularly serious concern. In China, it is common for families to rely on skip-generation child raising [49, 50], with a majority of children (70%) being predominantly cared for by their grandparents, and the remaining 30% being completely looked after by them [51]. However, the research conducted by Zhang Saiqun [52] revealed that a significant portion of grandparents (31.2%) felt unable to adequately care for a second child. Additionally, 47.8% of pregnant women reported not having enough time for child care, and 21.0% expressed negative sentiments towards hiring a nanny. Our own qualitative research findings were consistent with these observations. Pregnant women expecting a second child expressed concerns about their ability to manage the stress of pregnancy while balancing work responsibilities, the physical limitations of aging grandparents, and difficulties in finding suitable care arrangements.

To address these challenges, it is recommended that pregnant women proactively plan for child care well in advance. Furthermore, the acceptance of the second child by the first child is crucial. Chen Binbin et al. discovered that first children perceived the arrival of a sibling as a threat to their close bond with their mother and other family members [53]. They feared being replaced in their parents’ affections. Our own findings indicated that some first children often displayed anger upon learning about the impending arrival of a sibling. Therefore, it is important for pregnant women and other family members to learn effective strategies for facilitating the acceptance of the second child by the first child. Overall, by examining and addressing the various family factors associated with the stress experienced by women during their second pregnancy, we can better support the well-being of both mothers and their children.

Life events, career development, and workload were found to significantly contribute to the stress experienced during pregnancy. Specifically, pregnant women expressed concerns about their career advancement due to the arrival of a second child. It is important for the employer to acknowledge that the perinatal period of the second child requires additional time and effort to be devoted to the family, which may result in the mother being unable to continue in her current position and missing out on opportunities for promotion. Consequently, our research highlights the pressing need for immediate intervention to create more avenues for these women to pursue their professional goals.

### Strengths and limitations

The strength of our study lies in the implementation of a mixed-method approach to investigate factors associated with stress among pregnant women who are expecting their second child in the context of China’s two-child policy, a topic that has not been previously explored. To the best of our knowledge, this study is the first to provide comprehensive insights into the experience of stress within this specific population.

However, there are limitations in the current study that should be considered for future research. Firstly, it is important to acknowledge that participants’ responses may have been influenced by their mood at the time of completing the questionnaire. Furthermore, qualitative interviews were conducted only once during pregnancy, subsequent to the collection of quantitative data. Therefore, it would be beneficial to continuously monitor respondents throughout different stages of pregnancy, including early, mid, and late pregnancy. Secondly, it is crucial to note that the data used in this study were obtained from a third-level grade-A hospital, which may limit the representative of the study sample. To improve the study, it is advisable to implement continuous monitoring of respondents at various stages of pregnancy, while also expanding the participant pool to include a wider range of healthcare facilities.

## Conclusion

Our study used a mixed-method design to explore the factors that may influence the pregnancy stress of women with a second child. Most women are under low to moderate stress during pregnancy under China’s two-child policy. We discovered that a heavy family financial burden, lack of subjective support, voluntary fertility, the first child being a girl, women’s gender preference for a girl, and the three major themes of fertility factors, family factors and social factors were identified as factors affecting pregnancy stress. Therefore, interventions aimed at reducing or eliminating stress need to be strengthened by hospitals, families, and governments.

## Data Availability

The datasets produced and/or examined in this study are not accessible to the public due to restrictions imposed by the ethics committee. However, they can be obtained from the first author (Lijuan Zhang) upon a reasonable request.
